# Cumulative Effects of Weather on Stroke Incidence: A Multi-Community Cohort Study in Japan

**DOI:** 10.2188/jea.JE20090103

**Published:** 2010-03-05

**Authors:** Masatoshi Matsumoto, Shizukiyo Ishikawa, Eiji Kajii

**Affiliations:** Division of Community and Family Medicine, Centre for Community Medicine, Jichi Medical University, Shimotsuke, Tochigi, Japan

**Keywords:** meteorological factors, stroke, cerebral infarction, cohort studies, Japan

## Abstract

**Background:**

Although seasonal variation in stroke incidence has been reported, it is not known whether year-long exposure to particular meteorological conditions affects the risk of stroke independently of conventional cardiovascular risk factors.

**Methods:**

We conducted a cohort study involving 4849 men and 7529 women residing in 12 communities dispersed throughout Japan. Baseline data were obtained from April 1992 through July 1995. Follow-up was conducted annually to capture first-ever-in-life stroke events. Weather information during the period was also obtained for each community. Multilevel logistic regression analysis was conducted to evaluate the association between stroke incidence and each meteorological parameter adjusted for age, obesity, smoking status, total cholesterol, systolic blood pressure, diabetes, and other meteorological parameters.

**Results:**

Over an average of 10.7 years of follow-up, 229 men and 221 women had stroke events. Among women, high annual rainfall (OR per 1000 mm, 1.46; 95% confidence interval, 1.05–2.03), low average ambient temperature (OR per 1 °C, 0.79; 0.66–0.94), and number of cold days per year (OR per 10 days, 3.37; 1.43–7.97) were associated with increased risk of stroke incidence, independent of conventional risk factors. Among men, number of cold days (OR per 10 days, 1.07; 1.02–1.12) was associated with an increased risk of stroke incidence, but the association became nonsignificant after adjustment for other risk factors. Similar results were obtained for cerebral infarction and cerebral hemorrhage.

**Conclusions:**

Long-term exposure to some meteorological conditions may affect the risk of stroke, particularly in women, independent of conventional risk factors.

## INTRODUCTION

Various individual-level risk factors are known to affect the occurrence of stroke, including age, sex, hypertension, and diabetes, but very little is understood regarding the impact of environmental factors, such as the meteorological characteristics of a community.^1^^,^^2^ In several countries, stroke mortality and/or incidence rate increases in cold months; thus, exposure to low temperatures is suspected to be a risk factor for stroke.^3^^–^^6^ Increases in coagulation-related factors such as fibrinogen and factor VII,^7^ elevation of blood pressure,^8^^–^^10^ exacerbation of hemoconcentration,^11^^,^^12^ and increases in plasma lipids^13^ during the cold season have been advanced as mechanisms underlying the winter–stroke relationship. However, one study found no seasonal variation in stroke incidence rate,^14^ which leaves the issue unsettled.

Evaluating seasonal variation in stroke may reveal the role of meteorological conditions as triggers for stroke. However, this type of study cannot assess the cumulative effect of year-long exposure to a particular meteorological condition. In order to evaluate the long-term effects of weather on stroke incidence, it is necessary to conduct a prospective study based in multiple communities with varied weather conditions. Past studies have noted geographical variation in stroke incidence within a country. This variation could not be fully explained in terms of conventional risk factors such as age, sex, race, and blood pressure.^15^^–^^18^ However, none of these studies considered both weather conditions and the conventional risk factors in a single, appropriate statistical model. It remains unknown if weather has a cumulative impact on stroke even after adjusting for individual-level risk factors.

We conducted a cohort study based in 12 different communities scattered throughout Japan to determine if weather conditions in a community could predict first-ever-in-a-lifetime stroke incidence of the residents, independently of individual-level risk factors.

## METHODS

### Study population

The JMS Cohort Study began in 1992. Its primary objective was to clarify the relationship between potential risk factors and cardiovascular diseases in 12 of the 3238 rural municipalities (towns, villages, and cities) that were present in Japan in 1992.^19^ The baseline data of this cohort study were obtained from April 1992 through July 1995 and were collected as part of a national mass-screening program. In Japan, mass screening for cardiovascular diseases has been conducted since 1982, in accordance with the *Health and Medical Service for the Aged Act* of 1981. Eligible subjects for the mass-screening were residents aged 40 to 69 years in 11 of the 12 communities, and residents aged over 30 years in 1 community. Local government offices in each community issued invitations to eligible residents to attend the mass screening. As a result, 12 490 subjects (4913 males and 7577 females) participated in the study. Among these individuals, 112 had a past history of stroke and were excluded. Thus, 12 378 (4849 males and 7529 females) were included in the analysis. The total population of each community ranged from 430 to 19 227 (average, 6975). The participation rates for each community invited to the mass screening were as follows: 77% in Iwaizumi, 65% in Tako, 78% in Yamato, 69% in Kuze, 90% in Takasu, 79% in Wara, 88% in Sakuma, 30% in Hokudan, 40% in Sakugi, 66% in Okawa, 44% in Ainoshima, and 26% in Akaike.^20^ The overall participation rate was 65.4%. Written informed consent to participate in the study was obtained individually from all participants in the mass screening.

### Measurement of baseline variables

Body weight was recorded with the subject clothed; 0.5 kg in summer or 1 kg during the other seasons was subtracted from the recorded weight. Obesity was defined as a body mass index of ≥25 kg/m^2^. Blood samples were collected from all the participants; 5532 (44.3%) of these samples were drawn after overnight fasting. “Diabetic subjects” were defined as those with currently-treated diabetes, plasma glucose ≥126 mg/dL after overnight fasting, or casual blood glucose ≥200 mg/dL.

### Meteorological variables

Weather information for each community was obtained from the nearest observatory of the Japan Meteorological Agency, Ministry of Land, Infrastructure, Transport and Tourism. The distance from the center of a community and its observatory ranged from 0 to 28 km; the average was 11.3 km. The meteorological variables studied were annual cumulative rainfall (mm), mean daily temperature for a year (°C), mean daily temperature gradient (ie, difference between the mean maximum daily temperature and the mean minimum daily temperature) over a 1-year period (°C), number of days per year when the lowest ambient temperature was below 0 °C (“cold days”), and annual cumulative sunlight duration (hours). All the data were derived from the Agency’s web site.^21^ Data for each variable were obtained every year from 1995 through 2005, and the average value for the 11 years was used for the analysis.

Rainfall, temperature, and sunlight duration were measured in increments of 0.5 mm, 0.1 °C and 0.1 hours, respectively. The data on all variables were automatically recorded at each observatory every 10 minutes and were sent for statistical analysis (eg, calculation of daily and annual means) to the integrated data processing system at the Japan Meteorological Agency in Tokyo.

### Follow-up

Repeat examinations, which were also part of the national mass-screening program, were used to follow most subjects on an annual basis. Those examined were asked whether they had suffered a stroke after enrolling. Subjects who did not come to the screening examination were contacted by mail or phone. Those with a history of stroke were asked to identify the hospital in which they had been treated and when the condition was diagnosed. Medical records at hospitals in the study areas were checked to determine if study subjects were hospitalized for any reason, and public health nurses visited the subjects to obtain pertinent information when necessary. In total, 100% of the subjects were contacted. If an incident was suspected, images from computed tomography and/or magnetic resonance imaging were obtained for diagnostic confirmation of stroke.

### Diagnostic criteria

Diagnosis was determined independently by a diagnosis committee composed of 1 radiologist, 1 neurologist, and 2 cardiologists. A diagnosis of stroke was made on the basis of the presence of a focal and nonconvulsive neurological deficit, of clear onset, lasting for 24 hours or longer. Stroke subtypes were confirmed based on computed tomography and/or magnetic resonance imaging in all stroke cases except 2 (1.1%), whose images were unavailable, and in those cases for which the diagnosis was based only on medical records in local hospitals. The subtype classification was conducted according to the criteria of the National Institute of Neurological Disorders and Stroke.^22^

### Statistical analysis

Statistical analyses were carried out using SPSS^®^ for Windows, version 11.5 (SPSS Inc, Japan). Continuous variables were compared among communities using ANOVA. Categorical variables were compared using the chi-square test.

Our data consist of individual-level data nested within community-level data, and thus form a multilevel structure.^23^ Multilevel logistic regression analysis was conducted using the statistical package MLwiN, version 2.10 (Centre for Multilevel Modelling, University of Bristol, UK). The regression analysis was performed using a random intercept fixed slope model. For evaluating the associations between stroke incidence and each meteorological parameter, 3 separate models in each sex were generated. Model 1 was adjusted for age; model 2 was adjusted for age, obesity, current smoking status, total cholesterol, systolic blood pressure, and diabetes; and model 3 was adjusted for age, obesity, current smoking status, total cholesterol, systolic blood pressure, diabetes, and all the meteorological parameters. Because of strong collinearity between mean daily temperature and number of cold days, adjustment of these 2 parameters for each other was not conducted in model 3. The results are expressed as odds ratios (ORs) and 95% confidence intervals (CIs). The second-order, penalized, quasi-likelihood procedure was used for estimating the regression coefficients. Variance of the intercept in the 2-level null random intercept model without any explanatory variable was defined as the between-area variance. The intraclass correlation coefficient, ie, the ratio of the between-area variance and the total variance, was estimated as R/(R + 3.29), where R was the between-area variance.^23^ In all statistical tests, *P* < 0.05 was considered significant.

### Ethical approval

The study design and procedure were approved by the government of each community and by the Ethical Committee of Epidemiologic Research at Jichi Medical University.

## RESULTS

Among the eligible study subjects, 95 declined follow-up and 7 could not be followed. Thus, a total of 12 276 (4807 men and 7469 women) were followed (follow-up rate, 99.2%). The mean age at baseline was 55.2 years in men and 55.3 years in women. The mean duration of follow-up was 10.7 years.

**Figure 1.**
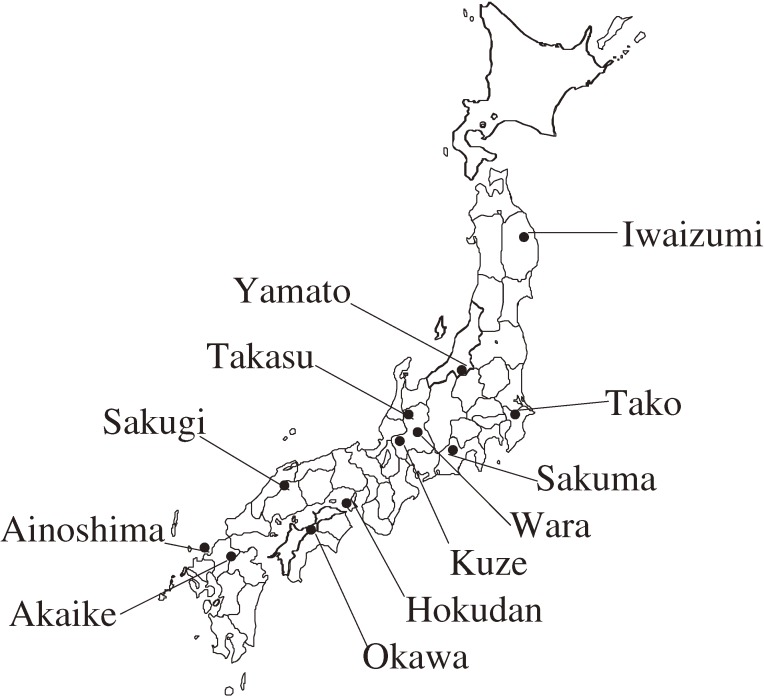
Distribution of participating communities

The geographic distribution of the 12 communities that participated in this study is shown in Figure 1. The individual-level and community-level variables that were investigated in each community are shown in Table 1. For all individual-level variables, significant differences among the communities were observed. The incidence rate of stroke ranged between 1.3 and 6.2 per 1000 person-years among the communities. The between-area variance for the incidence rate of total stroke was 0.180 (95% CI, −0.031 to 0.392) in men and 0.305 (−0.002 to 0.632) in women; the variance of cerebral infarction was 0.233 (−0.043 to 0.509) in men and 0.346 (−0.060 to 0.752) in women; and the variance of cerebral hemorrhage was 0.113 (−0.175 to 0.401) in men and 0.567 (−0.221 to 1.355) in women. The intraclass correlation coefficient for total stroke was 5.2% in men and 8.5% in women, the coefficient for cerebral infarction was 6.6% in men and 9.5% in women, and the coefficient for cerebral hemorrhage was 3.3% in men and 14.7% in women.

**Table 1.** Characteristics of participating communities

Level of variable	Variable	Iwaizumi		Tako		Yamato		Kuze		Takasu		Wara		Sakuma		Hokudan		Sakugi		Ohkawa		Ainoshima		Akaike		*P* value^a^
Individual	Subjects	1088		2837		2381		446		1403		1352		297		1119		386		205		133		629		
	Male (%)	383	(35.2)	1142	(40.3)	732	(30.7)	163	(36.5)	607	(43.3)	608	(45.0)	99	(33.3)	532	(47.5)	162	(42.0)	90	(43.9)	68	(51.1)	221	(35.1)	<0.001
	Age, y (SD)	57.2	(8.0)	55.5	(8.6)	51.7	(13.3)	57.5	(8.6)	53.6	(14.4)	58.2	(13.0)	62.6	(9.5)	54.1	(13.3)	57.0	(9.0)	58.6	(8.2)	59.2	(7.1)	54.7	(9.5)	<0.001
	Length of follow-up, y (SD)	10.7	(0.1)	11.4	(0.0)	10.3	(0.0)	11.3	(0.1)	11.8	(0.1)	10.9	(0.1)	10.0	(0.2)	10.7	(0.1)	10.7	(0.1)	9.4	(0.1)	11.1	(0.2)	10.9	(0.1)	<0.001
	Stroke (per 1000 person-years)	56	(4.8)	70	(2.2)	65	(2.6)	22	(4.4)	74	(4.5)	92	(6.2)	17	(5.7)	15	(1.3)	6	(1.5)	9	(4.7)	6	(4.1)	18	(2.6)	<0.001
	Cerebral infarction	30	(2.6)	48	(1.5)	36	(1.5)	15	(3.0)	54	(3.3)	58	(3.9)	14	(4.7)	9	(0.8)	3	(0.7)	6	(3.1)	5	(3.4)	12	(1.7)	
	Cerebral hemorrhage	20	(1.7)	15	(0.5)	17	(0.7)	6	(1.2)	10	(0.6)	20	(1.4)	1	(0.3)	4	(0.3)	1	(0.2)	3	(1.6)	1	(0.7)	4	(0.6)	
	Subarachnoid hemorrhage	6	(0.5)	7	(0.2)	12	(0.5)	1	(0.2)	10	(0.6)	14	(0.9)	1	(0.3)	2	(0.2)	2	(0.5)	0	(0.0)	0	(0.0)	2	(0.3)	
	Unclassified	0	(0.0)	0	(0.0)	0	(0.0)	0	(0.0)	0	(0.0)	0	(0.0)	1	(0.3)	0	(0.0)	0	(0.0)	0	(0.0)	0	(0.0)	0	(0.0)	
	Current Smoker (%)	185	(17.0)	549	(19.4)	556	(23.4)	111	(24.9)	316	(22.5)	283	(20.9)	59	(19.9)	301	(26.9)	72	(18.7)	33	(16.1)	36	(27.1)	134	(21.3)	<0.001
	Diabetes (%)^b^	47	(4.3)	53	(1.9)	105	(4.4)	28	(6.3)	56	(4.0)	67	(5.0)	15	(5.1)	20	(1.8)	14	(3.6)	10	(4.9)	2	(1.5)	25	(4.0)	<0.001
	Obesity (%)^c^	446	(41.0)	625	(22.0)	556	(23.4)	106	(23.8)	319	(22.7)	199	(14.7)	43	(14.5)	254	(22.7)	69	(17.9)	49	(23.9)	26	(19.5)	137	(21.8)	<0.001
	Systolic blood pressure, mm Hg (SD)	135.4	(22.3)	130.4	(17.2)	120.8	(19.0)	130.4	(20.0)	133.1	(22.5)	127.9	(21.3)	130.2	(22.3)	132.4	(21.7)	134.9	(21.3)	128.9	(24.6)	135.1	(23.1)	132.0	(21.8)	<0.001
	Total cholesterol, mg/dL (SD)	188.5	(32.5)	192.5	(34.5)	187.2	(34.9)	194.9	(36.2)	187.6	(35.3)	191.8	(32.7)	197.0	(35.1)	197.4	(36.3)	206.9	(33.1)	190.0	(34.9)	192.0	(36.3)	204.2	(36.9)	<0.001

Community	Annual rainfall (mm)	1104.1		1401.5		2317.3		2202.4		3314.4		2691.4		3165.9		1147.4		1505.6		3239.1		1580.5		1726.9		
	Temperature (°C)^d^	10.2		15.0		11.7		14.7		12.8		12.7		11.5		16.2		13.4		12.0		15.9		16.0		
	Temperature gradient (°C)^e^	10.2		8.9		8.2		10.8		8.6		10.1		9.1		8.1		10.3		9.2		8.2		9.4		
	Cold days (days)^f^	129.7		52.8		99.3		85.5		103.7		101.3		74.2		26.5		80.3		88.2		26.7		33.2		
	Annual sunlight duration (h)	1505.2		1816.9		1217.0		1564.9		1030.2		1515.3		1282.0		1992.9		1557.0		1335.0		1733.5		1861.2		

^a^ANOVA was used to compare continuous variables among communities; categorical variables were compared using the chi-square test.

^b^Those with currently-treated diabetes, plasma glucose ≥126 mg/dL after overnight fasting, or casual blood glucose ≥200 mg/dL.

^c^Those with a body mass index ≥25 kg/m^2^.

^d^Mean daily temperature per year.

^e^Gradient between the mean highest daily temperature and the mean lowest daily temperature per year.

^f^Number of days per year when the lowest temperature was lower than 0 °C.

The associations between meteorological parameters and total stroke incidence are shown separately for men (Table 2) and women (Table 3). In men, the number of cold days was associated with a significantly increased risk of total stroke in model 1, but not in models 2 or 3. In women, high rainfall, low ambient temperature, number of cold days, and short sunlight duration were associated with an increased risk of stroke in both model 1 and model 2. In model 3, in which meteorological parameters were adjusted for each other, the association of sunlight duration disappeared.

**Table 2.** Associations of meteorological factors with stroke incidence in men

	Model 1		Model 2		Model 3	
			
	OR	95% CI	OR	95% CI	OR	95% CI
Rainfall (per 1000 mm)	1.12	(0.84–1.49)	1.13	(0.81–1.57)	0.94	(0.59–1.49)
Temperature (°C)	0.92	(0.84–1.00)	0.93	(0.82–1.06)	1.05	(0.86–1.30)
Temperature gradient (°C)	1.23	(0.99–1.51)	1.24	(0.94–1.62)	1.29	(0.99–1.69)
Cold days (per 10 days)	**1.07**	(1.02–1.12)	1.50	(0.90–2.49)	0.96	(0.36–2.53)
Sunlight duration (per 1000 h)	0.56	(0.27–1.17)	0.57	(0.23–1.41)	0.37	(0.05–2.76)

Model 1: adjusted for age.

Model 2: adjusted for age, current smoking, blood pressure, diabetes, obesity, and total cholesterol.

Model 3: adjusted for age, current smoking, blood pressure, diabetes, obesity, total cholesterol, and meteorological variables.

Values in bold are statistically significant.

**Table 3.** Associations of meteorological factors with stroke incidence in women

	Model 1		Model 2		Model 3	
			
	OR	95% CI	OR	95% CI	OR	95% CI
Rainfall (per 1000 mm)	**1.59**	(1.17–2.17)	**1.48**	(1.13–1.95)	**1.46**	(1.05–2.03)
Temperature (°C)	**0.79**	(0.70–0.88)	**0.82**	(0.75–0.89)	**0.79**	(0.66–0.94)
Temperature gradient (°C)	1.16	(0.80–1.69)	1.09	(0.78–1.52)	1.04	(0.83–1.32)
Cold days (per 10 days)	**2.87**	(1.84–4.49)	**2.46**	(1.70–3.57)	**3.37**	(1.43–7.97)
Sunlight duration (per 1000 h)	**0.23**	(0.10–0.57)	**0.27**	(0.13–0.57)	2.11	(0.40–11.08)

Model 1: adjusted for age.

Model 2: adjusted for age, current smoking, blood pressure, diabetes, obesity, and total cholesterol.

Model 3: adjusted for age, current smoking, blood pressure, diabetes, obesity, total cholesterol, and meteorological variables.

Values in bold are statistically significant.

The relationships between meteorological factors and cerebral infarction are shown in Table 4 and Table 5. In men, no meteorological factor was associated with the incidence of cerebral infarction. In women, high rainfall, low ambient temperature, number of cold days, and short sunlight duration were associated with an increased risk of cerebral infarction in model 1 and model 2; however, the associations of rainfall and sunlight duration disappeared in model 3.

**Table 4.** Associations of meteorological factors with cerebral infarction in men

	Model 1		Model 2		Model 3	
			
	OR	95% CI	OR	95% CI	OR	95% CI
Rainfall (per 1000 mm)	1.22	(0.91–1.64)	1.27	(0.87–1.85)	1.01	(0.58–1.75)
Temperature (°C)	0.94	(0.83–1.06)	0.96	(0.82–1.12)	1.17	(0.92–1.47)
Temperature gradient (°C)	1.20	(0.92–1.58)	1.23	(0.88–1.72)	1.40	(0.95–2.06)
Cold days (per 10 days)	1.41	(0.88–2.26)	1.36	(0.71–2.61)	0.60	(0.19–1.85)
Sunlight duration (per 1000 h)	0.52	(0.25–1.08)	0.50	(0.18–1.45)	0.27	(0.03–2.93)

Model 1: adjusted for age.

Model 2: adjusted for age, current smoking, blood pressure, diabetes, obesity, and total cholesterol.

Model 3: adjusted for age, current smoking, blood pressure, diabetes, obesity, total cholesterol, and meteorological variables.

Values in bold are statistically significant.

**Table 5.** Associations of meteorological factors with cerebral infarction in women

	Model 1		Model 2		Model 3	
			
	OR	95% CI	OR	95% CI	OR	95% CI
Rainfall (per 1000 mm)	**1.59**	(1.15–2.20)	**1.40**	(1.07–1.83)	1.25	(0.95–1.66)
Temperature (°C)	**0.82**	(0.71–0.94)	**0.86**	(0.76–0.96)	**0.79**	(0.62–1.00)
Temperature gradient (°C)	1.09	(0.73–1.61)	1.01	(0.73–1.38)	0.94	(0.69–1.27)
Cold days (per 10 days)	**2.39**	(1.30–4.39)	**1.93**	(1.18–3.17)	**3.00**	(1.00–8.98)
Sunlight duration (per 1000 h)	**0.27**	(0.10–0.75)	**0.36**	(0.15–0.84)	3.35	(0.40–27.79)

Model 1: adjusted for age.

Model 2: adjusted for age, current smoking, blood pressure, diabetes, obesity, and total cholesterol.

Model 3: adjusted for age, current smoking, blood pressure, diabetes, obesity, total cholesterol, and meteorological variables.

Values in bold are statistically significant.

The association of each meteorological factor with cerebral hemorrhage is shown in Table 6 and Table 7. Again, in men, no meteorological factor was associated with the incidence of cerebral infarction. In women, low ambient temperature, number of cold days, and short sunlight duration were associated with an increased risk of cerebral hemorrhage in model 1 and model 2; however, the association of sunlight duration disappeared in model 3.

**Table 6.** Associations of meteorological factors with cerebral hemorrhage in men

	Model 1		Model 2		Model 3	
			
	OR	95% CI	OR	95% CI	OR	95% CI
Rainfall (per 1000 mm)	0.80	(0.55–1.17)	0.79	(0.53–1.19)	0.78	(0.38–1.60)
Temperature (°C)	0.93	(0.79–1.10)	0.93	(0.79–1.10)	0.92	(0.64–1.33)
Temperature gradient (°C)	1.23	(0.86–1.75)	1.28	(0.89–1.85)	1.09	(0.61–1.94)
Cold days (per 10 days)	1.39	(0.70–2.78)	1.41	(0.71–2.83)	1.74	(0.31–9.72)
Sunlight duration (per 1000 h)	0.91	(0.27–3.05)	1.06	(0.31–3.66)	0.54	(0.10–2.84)

Model 1: adjusted for age.

Model 2: adjusted for age, current smoking, blood pressure, diabetes, obesity, and total cholesterol.

Model 3: adjusted for age, current smoking, blood pressure, diabetes, obesity, total cholesterol, and meteorological variables.

Values in bold are statistically significant.

**Table 7.** Associations of meteorological factors with cerebral hemorrhage in women

	Model 1		Model 2		Model 3	
			
	OR	95% CI	OR	95% CI	OR	95% CI
Rainfall (per 1000 mm)	1.63	(0.82–3.23)	1.68	(0.80–3.50)	1.15	(0.76–1.73)
Temperature (°C)	**0.68**	(0.57–0.81)	**0.68**	(0.56–0.82)	**0.67**	(0.54–0.84)
Temperature gradient (°C)	1.19	(0.62–2.28)	1.14	(0.57–2.29)	0.86	(0.57–1.31)
Cold days (per 10 days)	**5.74**	(2.54–12.98)	**5.62**	(2.38–13.27)	**6.87**	(2.46–19.18)
Sunlight duration (per 1000 h)	**0.13**	(0.02–0.73)	**0.11**	(0.02–0.69)	0.77	(0.06–9.69)

Model 1: adjusted for age.

Model 2: adjusted for age, current smoking, blood pressure, diabetes, obesity, and total cholesterol.

Model 3: adjusted for age, current smoking, blood pressure, diabetes, obesity, total cholesterol, and meteorological variables.

Values in bold are statistically significant.

## DISCUSSION

The results of this study revealed that weather conditions may influence the risk of total stroke, ischemic stroke, and hemorrhagic stroke in women. The possibility that meteorological conditions are a trigger for stroke has been suggested in studies on seasonal variation of stroke incidence and stroke risk factors.^3^^–^^8^^,^^10^^,^^13^ The present study supports the possibility of a weather–stroke association by showing that stroke incidence may be affected by year-long exposure to particular weather conditions.

It has been suggested that low temperature is a trigger of stroke mortality and incidence, because stroke deaths and events increase in winter and decrease in summer.^3^^–^^6^ The results of this study indicate that low temperature and/or some other weather conditions not only triggered stroke, but also cumulatively affected the risk of stroke among populations with prolonged exposure to particular conditions. Elevated blood pressure and increased blood lipids due to cold temperatures have been hypothesized as possible mechanisms for the winter–stroke association.^8^^–^^10^^,^^13^ However, in this study, the relationship between low temperature and stroke was significant even after adjustment for blood pressure and total cholesterol. Thus, other biological reactions to cold, such as activation of coagulation-related factors,^7^ hemoconcentration,^11^^,^^12^ and increased blood viscosity,^12^ may explain the underlying pathophysiology that is responsible for this relationship.

Women were more vulnerable to meteorological factors than were men in terms of stroke incidence. Another study conducted in Japan also found that seasonal variation in stroke incidence was more apparent in women than in men.^6^ The adverse health effects of low temperature might be more prominent in women than in men.^24^^–^^26^ Although winter mortality is higher than in other seasons for both sexes,^3^ one study found that the gap was larger among women.^24^ The reason for this is unknown, but sex differences in vascular reaction to cold exposure might play a part. Vascular constriction due to cold exposure is greater in women than in men in both superficial and deep arteries.^25^^–^^28^ This augmented cold-induced vasoconstriction in women may be due to increased activity of adrenergic alpha2C-receptor, the expression of which is enhanced by estrogen.^26^ It is possible that greater vascular sensitivity to outdoor temperature among women is responsible for the larger impact of temperature on stroke.

Few studies have assessed the associations of rainfall and sunlight with stroke.^1^ The effect of rainfall may be confounded by ambient pressure. Low ambient pressure induces inflow of the surrounding air, causes ascending air current, and creates clouds and rain. Therefore, areas with lower ambient pressure tend to have more rain. A hypobaric environment is known to increase the concentrations of prothrombin and thrombin-antithrombin complex, and to enhance the activity of factor VIIa.^29^ In addition, low barometric pressure potentially increases the incidence of venous thromboembolism and pulmonary embolism.^30^^,^^31^ It may therefore be possible that low ambient pressure raised the incidences of stroke in women and created the positive association between high rainfall and stroke in this study.

One reason why the weather–stroke association has long been inconclusive is that there are few cohort studies of multiple communities that include meteorological factors as explanatory variables.^1^ In such studies that have been conducted, the community unit employed was very large, and individual-level variables and community-level variables were not treated in a single and appropriate statistical model.^16^ Our study employed a small geographical unit (average area of sampled communities, 161 square kilometers; average population, 6975). Moreover, weather information utilized in this study was obtained at a point near the center of each community (average distance, 11 kilometers). The study thus used data on meteorological phenomena to which each study participant was likely to be exposed. In addition, the study employed a multilevel model into which explanatory variables in different levels can be entered and analyzed for each variable’s independent contribution to stroke incidence.

One limitation of this study is that community characteristics other than weather were not controlled for. For example, population size and the degree of air pollution in the area may confound the results of this study.^32^^,^^33^ Also, the limited number of communities studied might have weakened the statistical power of the study to detect significant contributions of some weather parameters to stroke risk. Similarly, the fact that fewer men than women were enrolled in the study might have weakened the detection power of the analyses of men. Another limitation is that, although this was a prospective cohort study, statistical analysis was conducted with a logistic regression model in which the time scale was not taken into account. This may weaken our conclusion regarding the causal link between weather and stroke. Unfortunately, the Cox regression model is not widely used for multilevel analysis because it does not have a real intercept and becomes extremely complicated when used for multilevel analysis.^23^

The participation rate of the community population might have caused bias in our results. The communities with lower participation rates for baseline mass screenings, such as Hokudan, Sakugi and Akaike, tended to have lower incidence rates of stroke in this study. This may be because study participants in those communities are more health-conscious than those in other communities. At the same time, such communities tend to be located in more southern, ie, warmer, areas of Japan. We cannot deny the possibility that this resulted in a false positive association between low temperature and stroke in this study, a possibility that should be re-examined in future research.
